# Author Correction: Smart low interfacial toughness coatings for on-demand de-icing without melting

**DOI:** 10.1038/s41467-023-36927-w

**Published:** 2023-03-02

**Authors:** Zahra Azimi Dijvejin, Mandeep Chhajer Jain, Ryan Kozak, Mohammad H. Zarifi, Kevin Golovin

**Affiliations:** 1 Okanagan Polymer Engineering Research & Applications Laboratory, School of Engineering, University of British Columbia, Kelowna, BC V1V 1V7 Canada; 2 Department of Mechanical & Industrial Engineering, University of Toronto, Toronto, ON M5S 3G8 Canada; 3 Okanagan Microelectronics and Gigahertz Applications (OMEGA) Lab, School of Engineering, University of British Columbia, Kelowna, BC V1V 1V7 Canada; 4 Department of Materials Science & Engineering, University of Toronto, Toronto, ON M5S 3G8 Canada

**Keywords:** Sensors and biosensors, Polymers, Mechanical engineering, Surfaces, interfaces and thin films, Electrical and electronic engineering

Correction to: *Nature Communications* 10.1038/s41467-022-32852-6, published online 31 August 2022

The original version of this article contained errors in Eq. (1) and in Fig. 6b.

In Eq. (1), the values of the constant and coefficients were incorrectly given and the third term within the parentheses was missing the quadratic variable *T*^2^. The equation incorrectly read:

1 $$E={[10.4(1+0.107T+1.87\times {10}^{-4})]}^{-1}\pm 1\%$$

The correct form of Eq. (1) is:

1 $$E={[.104(1+1.07\times {10}^{-3}T+1.87\times {10}^{-6}{T}^{2})]}^{-1}\pm 1\%$$

Furthermore, the original version of this article contained an error in Fig. 6b, in which the ‘x’ and ‘y’ axes were incorrectly labelled as ‘Time (min)’ and ‘Resonant frequency (GHz)’, respectively. The correct version of Fig. 6b is:


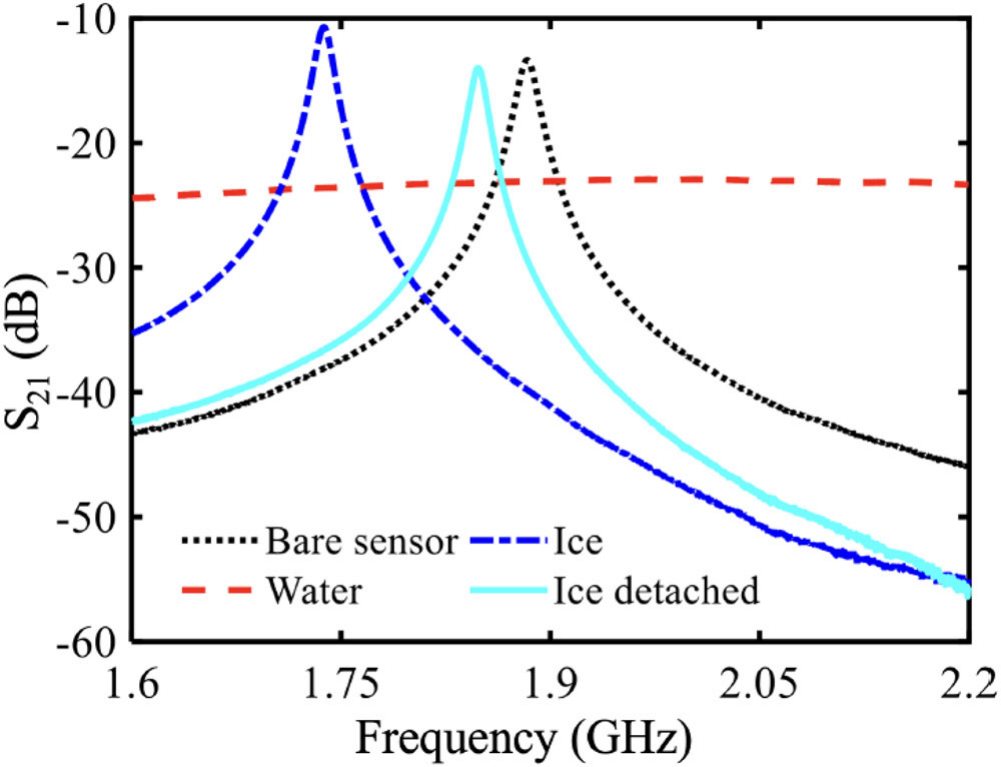


These have been corrected in both the PDF and HTML versions of the article.

