# Supplementary material for: Smart low interfacial toughness coatings for on-demand de-icing without melting
Source: Nat Commun. 2022 Aug 31;13:5119. doi: 10.1038/s41467-022-32852-6 (PMC9433454; doi:10.1038/s41467-022-32852-6)
Supplement: Supplementary file 1 — Supplementary Information [file 41467_2022_32852_MOESM1_ESM.pdf]

Supporting Information

Smart low interfacial toughness coatings for on-demand de-icing without melting

Zahra Azimi Dijvejin[1,2]*, Mandeep Chhajer Jain*[3]*, Ryan Kozak*[3]*, Mohammad H. Zarifi*[3]**, Kevin Golovin*[1,2,4]**

[1]Okanagan Polymer Engineering Research & Applications Laboratory, School of Engineering, University of British Columbia, Kelowna, BC, V1V 1V7, Canada

[2]Department of Mechanical & Industrial Engineering, University of Toronto, Toronto, ON, M5S 3G8, Canada

[3]Okanagan Microelectronics and Gigahertz Applications (OMEGA) Lab, School of Engineering, University of British Columbia, Kelowna, BC, V1V 1V7, Canada

[4]Department of Materials Science & Engineering, University of Toronto, Toronto, ON, M5S 3G8, Canada

*Corresponding author. E-mail Address: Kevin Golovin (Kevin.golovin@utoronto.ca) and Mohammad H. Zarifi (mohammad.zarifi@ubc.ca)

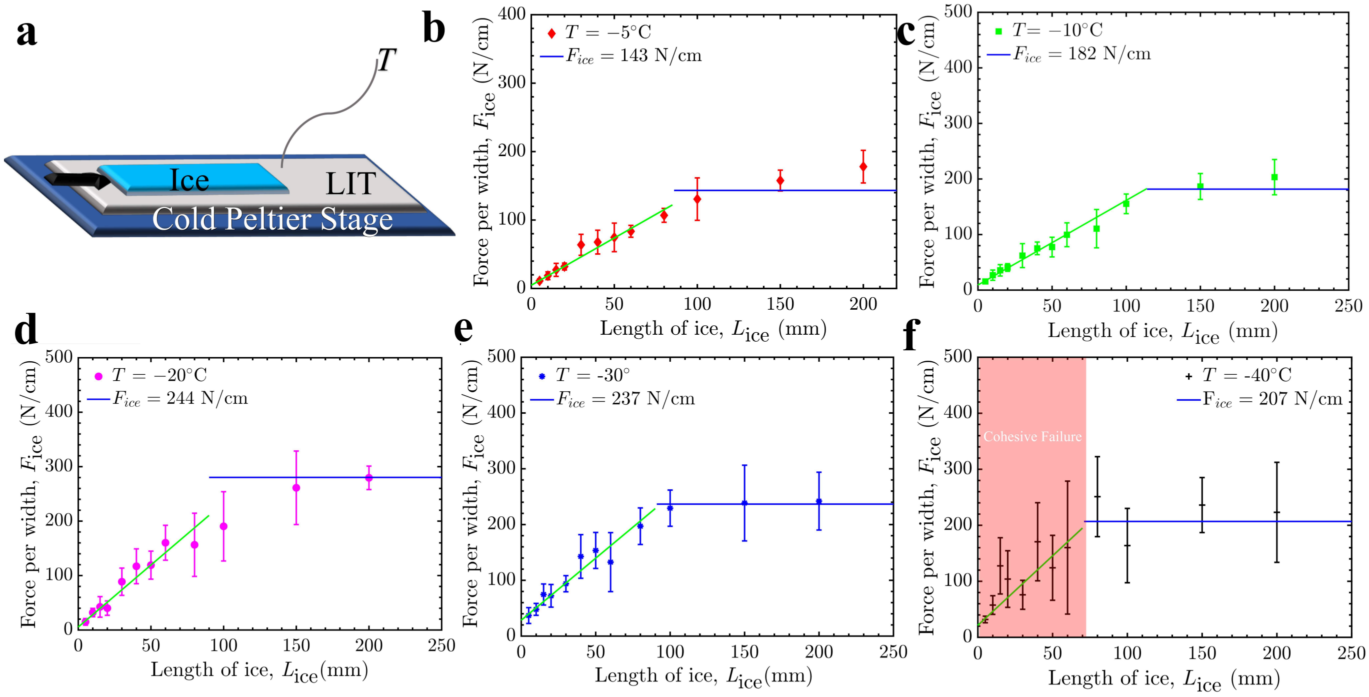

Supplementary Fig. S1 Effect of temperature on the interfacial properties between ice and the UHMW-PE material. a Schematic of ice adhesion measurement on the LIT-coated Al sheet. **b-f** The shear force per width required to remove the ice at different temperatures as a function of ice length: **b**, -5 °C, **c**, -10 °C, **d**, -20 °C, **e**, -30 °C, **f**, -40 °C. As the temperature was lowered, more force was required for ice removal. At -40 °C cohesive fracture was observed for the majority of the measurements in the strength-controlled fracture regime, though adhesive fracture was observed when toughness controlled the interfacial de-bonding. The parameters F_{ice} and T are representing the force to dislodge ice and the overall system temperature at the surface.

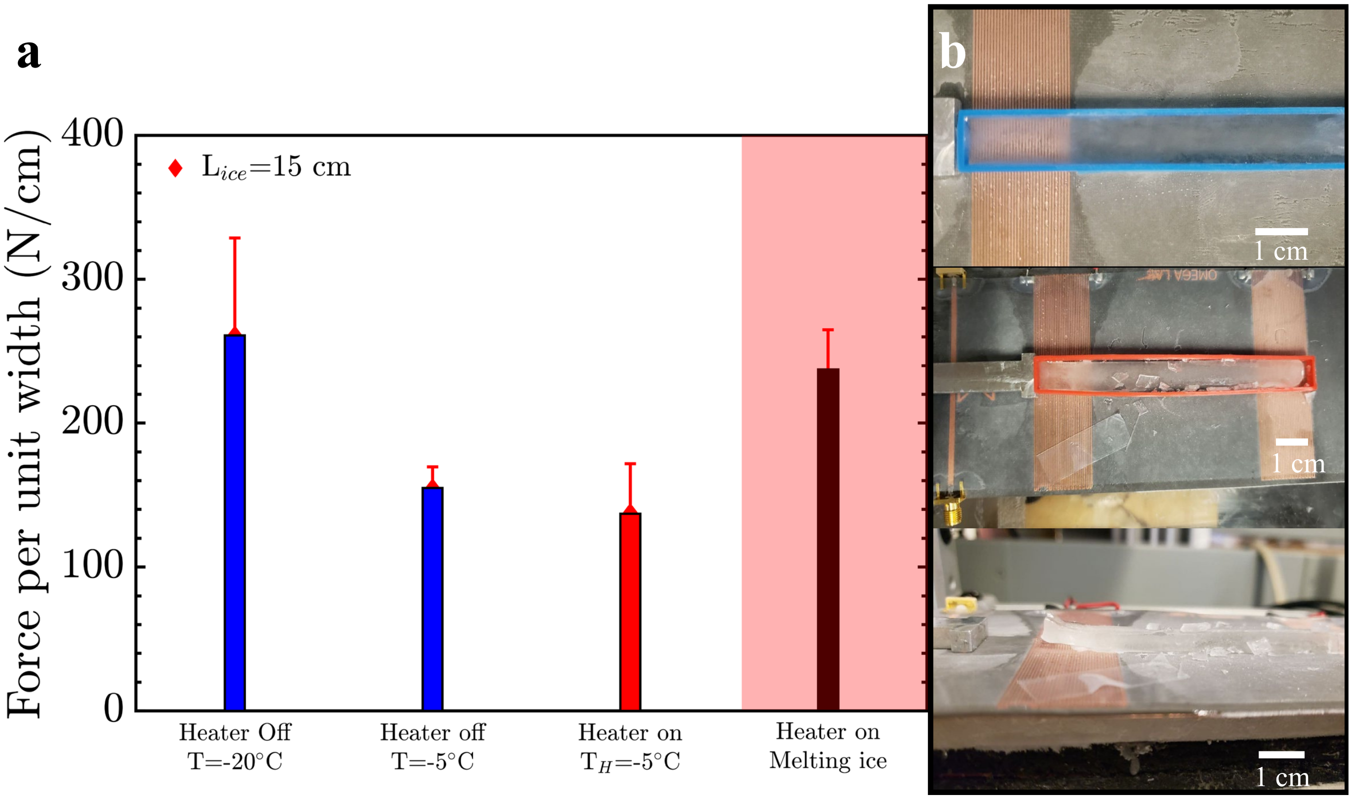

Supplementary Fig. S2 Effect of melting ice at LIT-coated heater on de-bonding force. a The force per width necessary to detach the ice from the LIT-coated heater for four different removal scenarios: (1) with the heater turned off at T = -20 °C; (2) with the heater turned off at T = -5 °C; (3) with the heater turned on such that T_H = -5 °C although the remainder of the surface was at T = -20 °C; and (4) allowing the heater to melt the ice directly above it, also at T = -20 °C. **b** Optical images of the ice when the heater was allowed to melt the ice directly above it. The top image shows the ice before applying the shear force. The middle image shows the shattered ice after cohesive failure. The bottom image shows the extent of the ice shattering with the 3D-printed mold removed. The parameters T and T_H are representing the overall system temperature and the temperature at the surface of the heater/LIT coating, respectively.

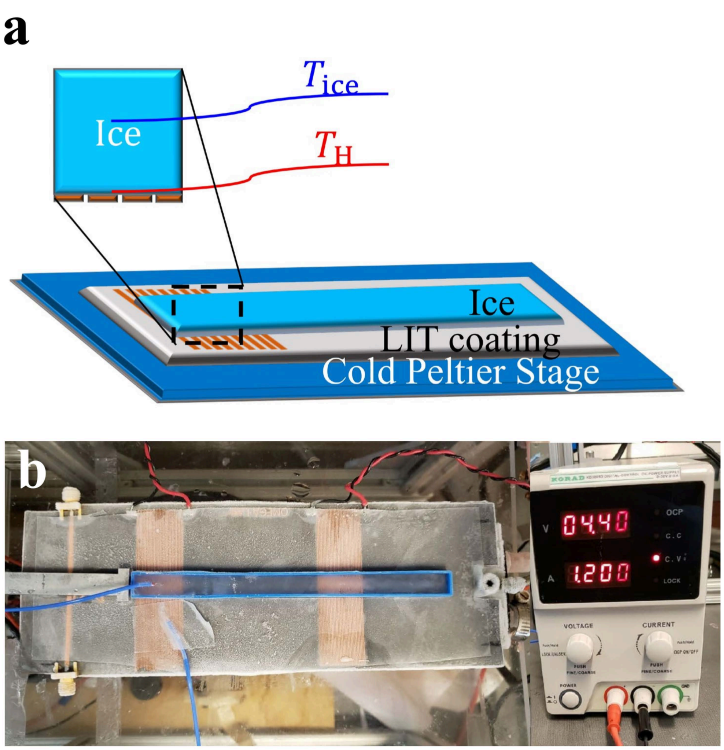

Supplementary Fig. S3 De-icing force measurements. a Schematic setup for monitoring the ice and coating temperature during de-icing. **b** Heater operating at 4.4 V using a power supply. At the target temperature of the LIT coating above the heater, T_H, the force probe impacts the ice in shear in order to remove it from the surface. The parameters T and T_H are representing the overall system temperature and the temperature at the surface of the heater/LIT coating, respectively.

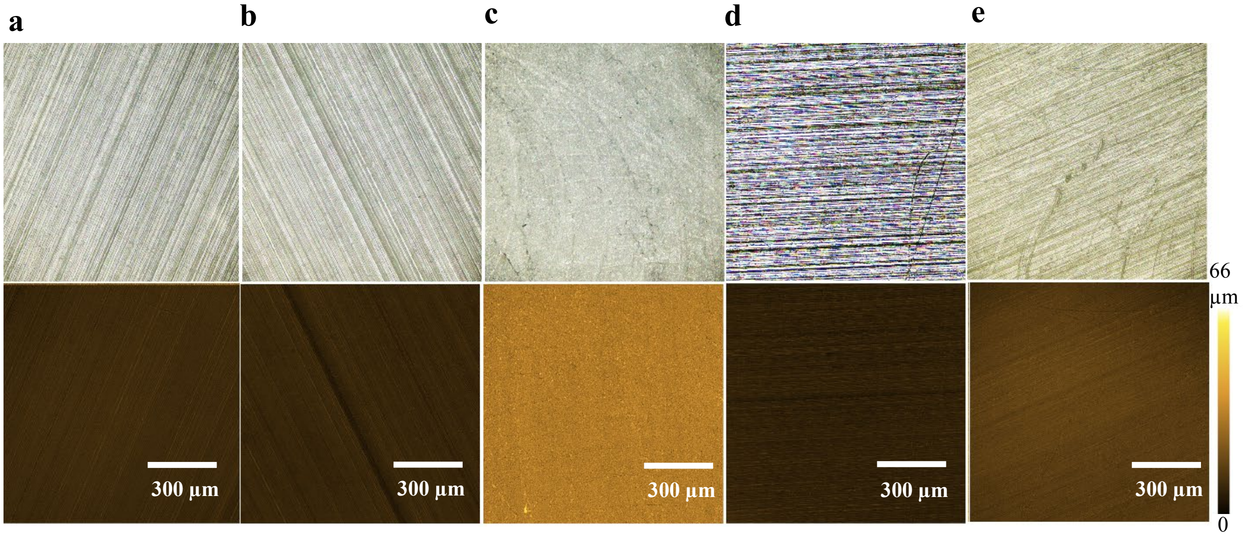

Supplementary Fig. S4 Optical and topography images after durability tests. a UHMW-PE sheet before exposure (S_q = 1.53 ± 0.05 µm) and after **b** Chemical contamination by acetone (S_q = 1.92 ± 0.08 µm); **c** mechanical abrasion (S_q = 3.4 ± 0.3 µm); **d** Cyclic icing/de-icing (S_q = 1.4 ± 0.2 µm); and **e** outdoor exposure (S_q = 1.5 ± 0.3 µm). The parameter of S_q is the Root Mean Square Roughness. (Top images are optical and bottom images are topography heightmaps).

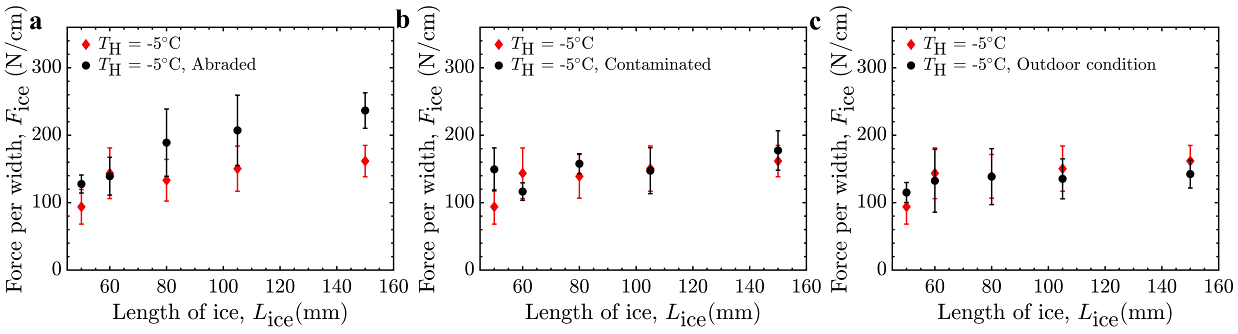

Supplementary Fig. S5 De-icing force versus ice length of LIT material after durability characterization. **a** After abrasion with 800 grit sandpaper for 15 minutes; **b** After contamination with acetone residue; **c** After exposure to the outdoors for 3 weeks in Toronto, ON, Canada. The parameters F_{ice} and T_H are representing the force to dislodge ice and the temperature at the surface of the heater/LIT coating, respectively.

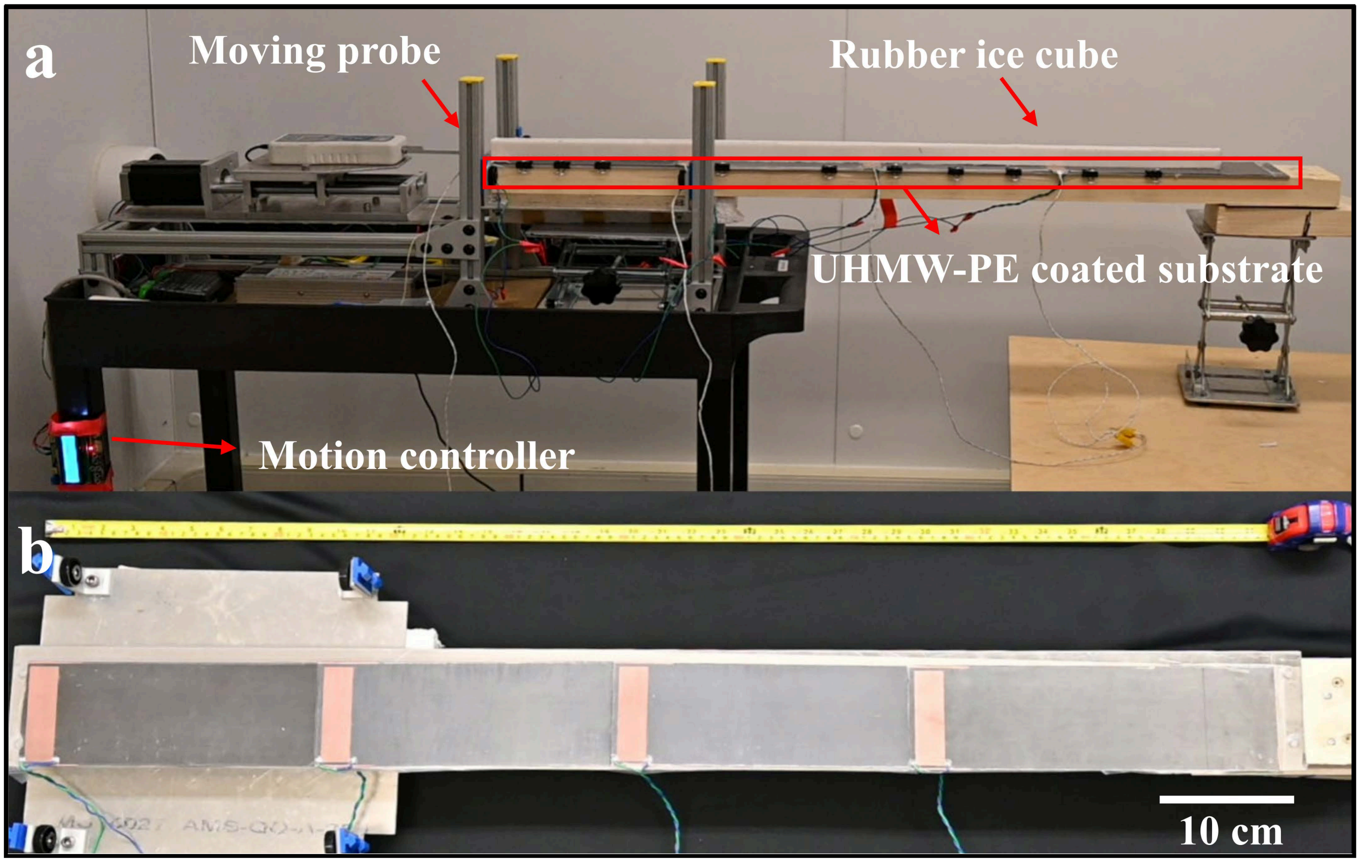

Supplementary Fig. S6 De-icing force measurements in walk-in freezer at -20°C. a The ice adhesion setup involving the UHMW-PE coated substrate, heaters, ice, and mechanical detachment instrumentation; **b** Top view of LIT-coated substrate decorated with four heaters with dimensions of 2.54 cm × 8 cm.

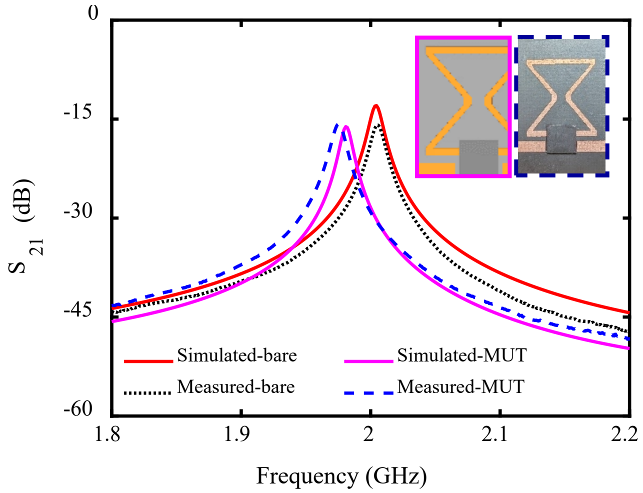

Supplementary Fig. S7 Simulated and measured transmission coefficient (*S_{21}*) for the bare sensor and in the presence of a material under test (MUT). A sample under test with a permittivity of 2.2 (to simulate ice), loss tangent of 0.0009, and dimensions of 5 × 5 × 0.79 mm was introduced in the vicinity of the sensor. The sensor's simulated and measured responses have a resonant frequency, resonant amplitude, and quality factor of 2.005 GHz, - 12.95 dB and 205, and 2.182 GHz -15.92 dB, and 167, respectively. The interaction between the material and the sensor's electromagnetic fields caused a shift in the resonant frequency and resonant amplitude by 30 MHz and -2.8 dB, respectively. The simulation results closely follow with the measured response of the sensor, with the slight difference between the two attributed to errors in the fabrication process and soldering of the Sub-Miniature A (SMAs), neither of which were considered in the simulation. The parameter S_{21} shows the measurements of power transmitted from port 1 to port 2.

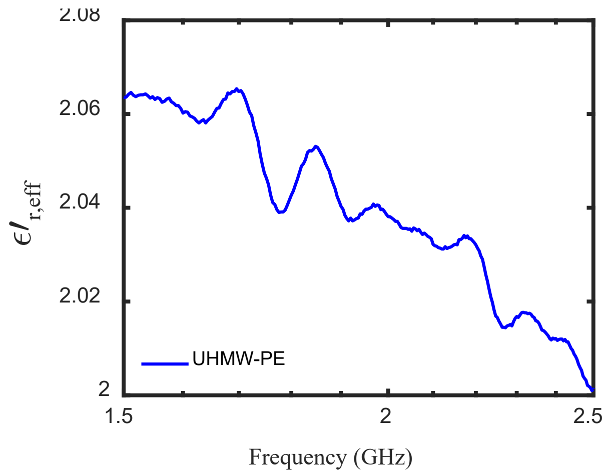

Supplementary Fig. S8 Effective relative permittivity ($\boldsymbol{\varepsilon}'_{r,}$) of UHMW-PE measured using a N1501A dielectric probe kit. The $\boldsymbol{\varepsilon}'_{r,eff}$ of LIT coating changes by increasing resonant frequency.

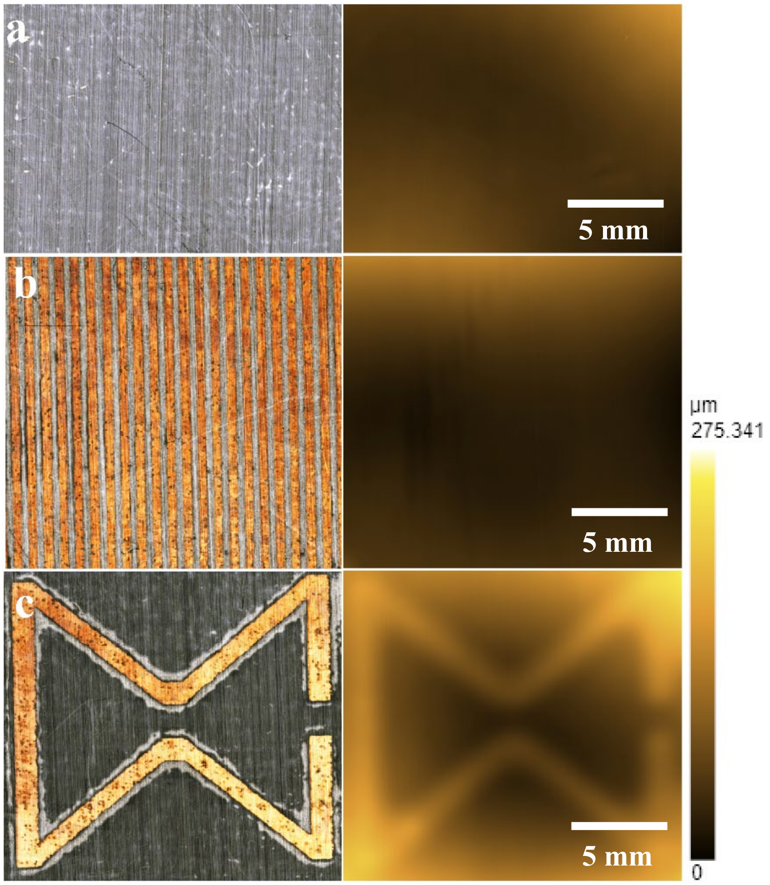

Supplementary Fig. S9 Optical and topography images. a UHMW-PE sheet (Root-mean-squared roughness, S_q = 1.53 µm); **b** Mini PCB heater covered by the UHMW-PE material (S_q = 1.51 µm); **c** The microwave sensor covered by the UHMW-PE coating (S_q = 1.50 µm).
