# Supplementary material for: Smart low interfacial toughness coatings for on-demand de-icing without melting
Source: Nat Commun. 2022 Aug 31;13:5119. doi: 10.1038/s41467-022-32852-6 (PMC9433454; doi:10.1038/s41467-022-32852-6)
Supplement: Supplementary file 3 — Description of Additional Supplementary Files [file 41467_2022_32852_MOESM3_ESM.pdf]

Description of Additional Supplementary Files

File Name: Supplementary Movie 1
Description: Interfacial crack growth observed during the application of a de-icing force/width of 114 N/cm to a 50 cm long piece of ice (L >> L_c).
